# Computational Analysis of Host–Pathogen Protein Interactions between Humans and Different Strains of Enterohemorrhagic *Escherichia coli*

**DOI:** 10.3389/fcimb.2017.00128

**Published:** 2017-04-19

**Authors:** Tungadri Bose, K. V. Venkatesh, Sharmila S. Mande

**Affiliations:** ^1^Bio-Sciences R&D Division, TCS Innovation Labs, Tata Consultancy Services LimitedPune, India; ^2^Department of Chemical Engineering, Indian Institute of Technology BombayMumbai, India

**Keywords:** *Escherichia coli* serotype O157:H7, host–pathogen interaction, protein interaction network, surface and secreted proteins, host colonization index

## Abstract

Serotype O157:H7, an enterohemorrhagic *Escherichia coli* (EHEC), is known to cause gastrointestinal and systemic illnesses ranging from diarrhea and hemorrhagic colitis to potentially fatal hemolytic uremic syndrome. Specific genetic factors like *ompA, nsrR*, and *LEE* genes are known to play roles in EHEC pathogenesis. However, these factors are not specific to EHEC and their presence in several non-pathogenic strains indicates that additional factors are involved in pathogenicity. We propose a comprehensive effort to screen for such potential genetic elements, through investigation of biomolecular interactions between *E. coli* and their host. In this work, an *in silico* investigation of the protein–protein interactions (PPIs) between human cells and four EHEC strains (viz., EDL933, Sakai, EC4115, and TW14359) was performed in order to understand the virulence and host-colonization strategies of these strains. Potential host–pathogen interactions (HPIs) between human cells and the “non-pathogenic” *E. coli* strain MG1655 were also probed to evaluate whether and how the variations in the genomes could translate into altered virulence and host-colonization capabilities of the studied bacterial strains. Results indicate that a small subset of HPIs are unique to the studied pathogens and can be implicated in virulence. This subset of interactions involved *E. coli* proteins like YhdW, ChuT, EivG, and HlyA. These proteins have previously been reported to be involved in bacterial virulence. In addition, clear differences in lineage and clade-specific HPI profiles could be identified. Furthermore, available gene expression profiles of the HPI-proteins were utilized to estimate the proportion of proteins which may be involved in interactions. We hypothesized that a cumulative score of the ratios of bound:unbound proteins (involved in HPIs) would indicate the extent of colonization. Thus, we designed the Host Colonization Index (HCI) measure to determine the host colonization potential of the *E. coli* strains. Pathogenic strains of *E. coli* were observed to have higher HCIs as compared to a non-pathogenic laboratory strain. However, no significant differences among the HCIs of the two pathogenic groups were observed. Overall, our findings are expected to provide additional insights into EHEC pathogenesis and are likely to aid in designing alternate preventive and therapeutic strategies.

## Introduction

*Escherichia coli* O157:H7 has emerged as an important zoonotic pathogen. In recent years, different strains belonging to the O157:H7 serotype of *E. coli* has been reported to be associated with disease outbreaks in U.S., Canada, Japan, and UK (Karmali, 1989; Zhang et al., 2007; Tildesley et al., 2012). It is one of the most prominent classes of enterohemorrhagic *Escherichia coli* (EHEC) and is known to cause gastrointestinal and systemic illnesses (Karmali, 1989; Zhang et al., 2007). In humans, the illness caused by EHEC ranges from diarrhea and hemorrhagic colitis to the more severe and potentially fatal hemolytic uremic syndrome (Karmali, 1989; Karch et al., 2005).

Although the primary habitat of *E. coli* O157:H7 is the ruminant gut, it has also been observed to colonize the human gastrointestinal tract. The infection resulting due to its colonization has been reported to be more severe in humans as compared to that in cattle (Zhang et al., 2010). Consequently, attempts have been made to understand the spatiotemporal nature of immune response in cattle under the influence of *E. coli* O157:H7 infection (Tildesley et al., 2012). The role of bacterial factors, like the type III secretion system, that are essential for colonization and persistence inside the cattle GI tract, have also been extensively studied (Zhang et al., 2010; Tildesley et al., 2012). However, the interplay between the host and the pathogenic factors are poorly understood. A few studies have also reported the effect of *E. coli* O157:H7 colonization on the human immune system (Li et al., 2000; Ho et al., 2013a,b). Studies have suggested the roles of specific bacterial proteins like OmpA (Outer membrane protein A) in *E. coli* (EHEC) pathogenesis (Torres et al., 2006). In addition, the role of NsrR (nitric oxide sensor nitrite-sensitive repressor) in the positive regulation of the *LEE* (Locus of Enterocyte Effacement) genes has also been solicited (Branchu et al., 2014). However, these genetic factors are not specific to EHEC and their presence in genomes of several non-pathogenic strains indicates that additional factors are likely to be involved in manifestation of pathogenicity by the EHEC strains. Thus, a comprehensive effort would be required to screen for (and model interplay between) potential genetic elements which may contribute toward successful propagation and virulence of EHEC strains inside the human body.

In order to understand the mechanism of bacterial association with its host, it is important to study protein–protein interaction (PPI) between the bacterial and the host proteins. Several researchers in the past have explored the avenue of studying “host–pathogen PPI” (HPI) in order to decipher the cross-talks between the host and the pathogen during the course of an infection (Evans et al., 2009; Tyagi et al., 2009; Wuchty, 2011; Zhou et al., 2014). Interestingly, earlier reports have indicated variations in the magnitude of immune responses among cattle calves infected with different strain of *E. coli* (EHEC; Corbishley et al., 2014). A comprehensive study comparing the differences in HPI patterns involving a host and various strains of *E. coli* (EHEC) is unavailable. Minor differences among the pathogenic determinants have also been reported in different strains of *E. coli* (EHEC) which affect the cattle calves (Corbishley et al., 2014). Therefore, inspection of the PPIs among host and different strains of *E. coli* could be helpful in decoding the probable tactics adopted by the bacteria to counter host defensive strategies. As one would expect, the dynamics of the colonization within the host GI tract (and the subsequent infection) would depend on interactions between the host and the pathogen proteins. In other words, the response of the host (human or cattle) would be governed by not only the genomic differences among bacterial strains, but also the host proteins which interact with the bacterial proteins. It is also well-documented that certain lineage specific variations exist among the genomes of different strains of *E. coli* O157:H7. These include the encoded Shiga toxins, the arginine translocation system, adhesion factors, and other mobile genetic elements (Zhang et al., 2010; Eppinger et al., 2011). Further, *E. coli* O157:H7 strains belonging to such lineages differ in their distribution across human and cattle hosts (Zhang et al., 2010). A comparative analysis of the HPIs would help in understanding probable mechanisms of host (human or cattle) invasion by various strains of *E. coli* (EHEC).

Most of the reported HPI studies have focused on understanding interactions of a specific pathogenic strain with its host (Evans et al., 2009; Tyagi et al., 2009; Wuchty, 2011; Zhou et al., 2014). In order to understand interactions of various strains of *E. coli* with human cells, we have developed a strategy for predicting HPIs between the host and multiple strains of *E. coli* using publicly available information (completely sequenced genomes as well as intra-species PPI data). A comparative analysis involving four EHEC strains of *E. coli* (viz., EDL933, Sakai, EC4115, and TW14359) and a non-pathogenic laboratory strain of *E. coli* (K-12 MG1655) was performed. Notably, while two of the pathogenic strains (EDL933 and Sakai) belonged to lineage I which are equally distributed among humans and cattle, EC4115 and TW14359 belonged to lineage I/II, which are known to share genetic and physiological characteristics intermediate to those of strains from lineages I and II (Zhang et al., 2010; Eppinger et al., 2011). Results of the present study indicate that a small subset of HPIs is unique to the studied pathogens which may contribute toward expression of virulent phenotypes. In addition, clear differences in lineage and clade specific HPI profiles could also be identified. Furthermore, we utilized gene expression profiles of the HPI–proteins to estimate the proportion of proteins which may be involved in the interactions. Based on the ratios of bound protein to total protein concentrations (involved in different HPIs), we designed a metric (called the Host Colonization Index or HCI) to determine the host colonization potential of the *E. coli* strains. We observed that pathogenic strains of *E. coli* had higher HCI as compared to non-pathogenic laboratory strain, thereby, re-emphasizing the vital role of HPIs in bacterial pathogenesis. Overall, our findings are expected to provide additional insights into EHEC pathogenesis and aid in designing alternate preventive and therapeutic strategies.

## Results

### Protein–protein interactions involving human cells and *E. coli*

The inter-species interlogs method (details in Materials and Methods Section) was employed to predict host–pathogen interactions (HPIs) between proteins from humans and those from the five studied strains of *E. coli*. Supplementary Table 1 lists the predicted HPIs corresponding to the five studied *E. coli* strains. The interactomes contained 762–783 HPIs and were comprised of 165–171 human proteins and 118–122 *E. coli* proteins. The HPI networks obtained between human and all the studied strains of *E. coli* were observed to have a single major sub-network (comprising of over 125 proteins) and several small sub-networks, each comprising of 2–25 interacting protein.

In order to identify key host as well *E. coli* proteins that are likely to play important roles during pathogenesis, graph properties like degree and betweenness centralities were analyzed from the obtained PPI networks. Degree, a measure of the number of links that any node in the network (in this case, a protein from either host or *E. coli*) shares with the other nodes, is a direct indicator of the importance of that protein in the host–pathogen cross-talks. It was observed that, while the average degree of the networks ranged between 5.2 and 5.4, the degree distribution of some *E. coli* proteins like the L-cystine-binding protein (FliY), UDP-sugar hydrolase (UshA), acyl-CoA thioesterase I (TesA), ferrienterobactin-binding periplasmic protein (FepB), glutathione peroxidase (BtuE), ferric hydroxamate uptake protein D (FhuD), and glutamine-binding periplasmic protein (GlnH), and some human proteins of the ATP-binding cassette like ABCB5, ABCA6, ABCB1, ABCB4, ABCB9, ABCA10, ABCA12, ABCA9, ABCB6, and ABCB8 were appreciably higher (≥20). On the other hand, betweenness of a node (protein) was calculated for measuring the number of shortest paths passing through that node. As expected, most of the human and *E. coli* proteins which demonstrated high degree centralities also had high betweenness values. However, it was interesting to note that some human proteins like formyltransferase (ATIC), thymidylate synthetase (TYMS), glutathione S-transferase zeta 1 (GSTZ1), pyruvate dehydrogenase (PDPR), aminomethyltransferase(AMT), cyctathione gamma lyase (CTH), glutamic oxaloacetic transaminases 1 and 2 (GOT1 and GOT2) had high betweenness centralities, in spite of low degree distributions. The gene encoding one of these proteins, namely GSTZ1, has been previously reported to have a staggering 20-fold over-expression in *E. coli* infected murine models (Motley et al., 2004). This observation indicates the likely involvement of the identified human proteins in *E. coli* mediated host responses. Furthermore, two *E. coli* proteins, namely, sulfate-binding protein (Sbp) and thiosulfate sulfurtransferase (YnjE) demonstrated high betweenness centralities, in spite of their relatively low degree distributions. Homologs of YnjE have previously been implicated for the virulent properties of *Salmonella enterica* serovar, when studied for systemic infection in a mouse model (Wallrodt et al., 2013). The above observations further indicate a role of these proteins during bacterial colonization in the host gut.

### Comparative analysis of protein interactions between human cells and different strains of *E. coli*

**Figure 1:**
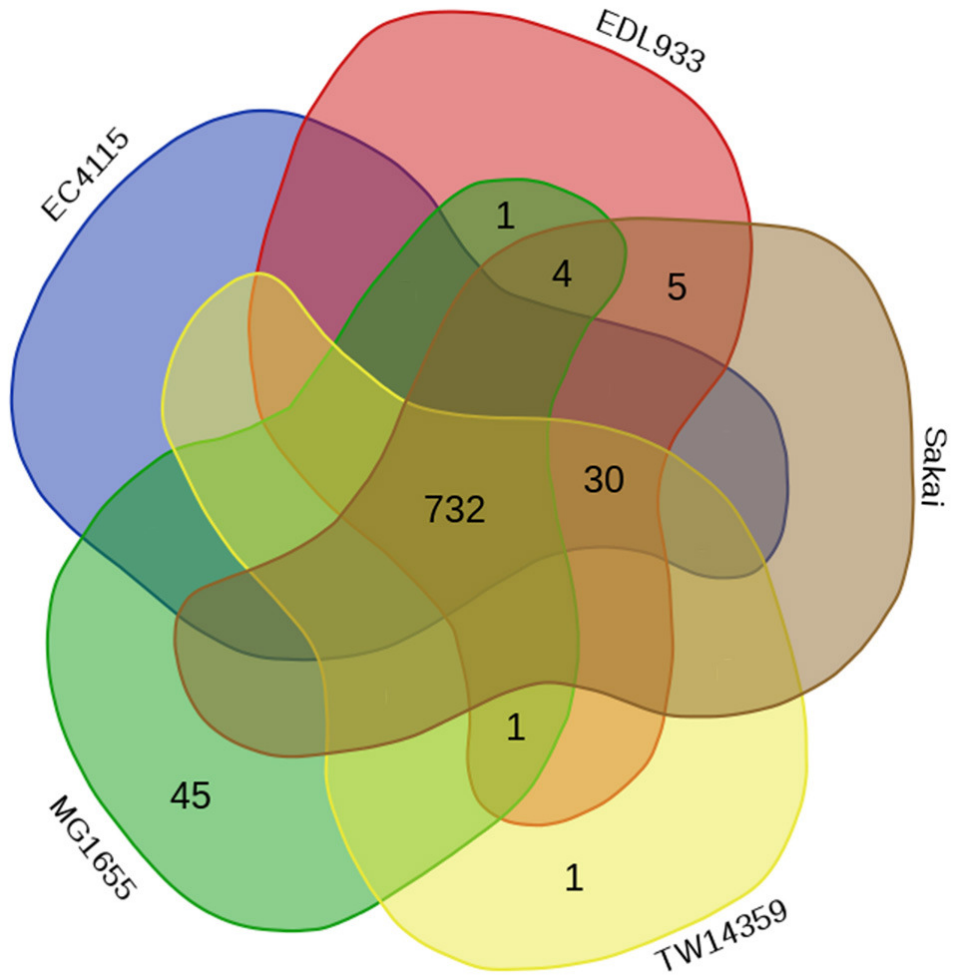
**Euler diagram representing the number of HPIs involving human proteins and those from the different studied ***Escherichia coli*** strains**.

The *E. coli* O157:H7 strains considered in the current study are known to be associated with disease outbreaks of varying magnitudes in different parts of the world (Zhang et al., 2007; Deng et al., 2011; Eppinger et al., 2011). It may be assumed that the interactome profile of these *E. coli* strains with the human cells would have a significant bearing on the nature of infection caused by these strains. It is therefore important to understand how genomic variations of these strains could affect their (host–pathogen) interactome profiles. Furthermore, whether and how the observed variations in the interactome profile pertaining to these strains translate into altered virulent potentials also remains to be deciphered. In order to evaluate whether proteins from these studied *E. coli* strains interact differently with the human cells, the obtained HPIs between host and different *E. coli* strains were compared. Results of the comparison indicated that although the majority of the interactions between host proteins and those in various *E. coli* strains were common to both pathogenic as well as non-pathogenic strains, 30 interactions were observed to be unique to the four pathogenic strains (Figure 1). In contrast, 45 HPIs were noted to be present only in the interactome corresponding to the non-pathogenic *E. coli* strain (Figure 1). The above observation suggests distinct differences between the HPI pattern involving human cells pathogenic and non-pathogenic strains of *E. coli*.

#### Human–*E. coli* protein interactions common to all studied strains

The majority (over 90%) of the PPIs were seen to be ubiquitously present in the interactome data involving human cells and the five studied strains of *E. coli*. Literature evidence supporting the biological basis for several of the identified HPIs could be obtained. For example, human nitric oxide synthase (NOS3) was found to interact with CysP of *E. coli*. Interestingly, NOS3 is involved in the synthesis of peroxynitrite (ONOO^−^), a known agent for innate immunity against enteric pathogens (Henard and Vázquez-Torres, 2011). Further, the level of expression of *cysP* and other related genes (in *E. coli*) have been reported to increase by 4- to 6-fold on exposure to peroxynitrite (McLean et al., 2010). Similarly, the observed interaction between host GGTLC1 (gamma-glutamyltransferase light chain) and the pathogenic *gadB* (glutamate decarboxylase) is interesting since *E. coli* GadA and GadB have previously been found to be responsible for the pathogenic ability to withstand the highly acidic environment within the human gut (Ma et al., 2002). In yet another HPI, the predicted interaction between *E. coli* KatG and KatE with superoxide dismutase (SOD2) of the host could find a support from earlier biological findings. While human SOD2 is devised for neutralizing intracellular supderoxide (${\text{O}}_{2}^{-}$) stress by producing hydrogen peroxide (H_2_O_2_), the bacterial KatG and KatE enzymes function by detoxifying the H_2_O_2_-induced stress in *E. coli* (Jung and Kim, 2003).

Several predicted interactions between human and *E. coli* proteins were found to involve bacterial chaperones and other proteins which aid the bacteria in protein folding. This finding is significant since earlier reports have indicated the potency of bacterial chaperones to act as virulence factors, influence host cell–cell signaling, and even promote apoptosis (Henderson et al., 2006). For instance, *E. coli* PpiA was observed to interfere with the functioning of several human proteins including FK506 binding protein 2 (FKBP2), tyrosine phosphatase (PRL), calnexin (CANX), squamous cell carcinoma antigen (SART3), basigin (BSG), transcriptional repressor protein YY1, and prolactin receptor (PRLR). While most of these human proteins were found to be associated with the endoplasmic reticulum, the *E. coli* PpiA, a chaperone, is known to play a vital role in protein folding and is essential for bacterial growth (Justice et al., 2005). DsbA, yet another bacterial protein which is involved in periplasmic disulfide bond formation, was observed to interact with the human intestinal-type alkaline phosphatase (ALPI). ALPI was further observed to interact with key bacterial proteins like SecA (essential for protein export), FolB and FloE (involved in folate biosynthesis), CreC (member of two-component signal transduction machinery), and GlgA (glycogen synthase). Interestingly, it has earlier been proposed that the intestinal alkaline phosphatases have the ability to detoxify (bacterial) lipopolysaccharide and hence, prevent bacterial invasion across the gut mucosal barrier (Goldberg et al., 2008). The identified PPIs involving human ALPI demonstrates that ALPI probably interferes with a larger array of previously unreported bacterial pathways and is important for host defense against an invading *E. coli*.

In the predicted HPI models, bacterial proteins like FhuD and FhuA were observed to interact with various members of the human ABC transporter family like ABCC6, ABCC8, and ABCG8 in all strains of *E. coli*. Bacterial FhuD and FhuA are components of the *fhu* operon which are known to be essential for utilization of ferric siderophores, thereby aiding the pathogen in iron acquisition (Mademidis and Köster, 1998). Given the low availability of iron/heme *in vivo*, the interaction of FhuD and FhuA with the human ABC transporters may be crucial for the survival of the pathogen.

In spite of the subtle differences in the HPI profiles, the majority of interactions were observed to be commonly present in the interactome data corresponding to the different studied strains of *E. coli*. While some of these interactions appeared to be involved in the mechanism by which the *E. coli* intrude into the host, others were representations of the host's immune response to counter an invading pathogen. This result indicates that the mechanism by which *E. coli* attaches to the host epithelium and subsequently colonizes the host gut is largely ubiquitous.

#### Human–*E. coli* protein interactions common to all pathogenic studied strains

Although most of the PPIs were common to the interactome data involving both pathogenic and non-pathogenic strains of *E. coli*, a small subset of HPIs (30 interactions) were specifically observed to be associated with the pathogenic strains (Figure 1). Such HPIs include *E. coli* EivG (type III secretion apparatus protein), ChuT (associated with heme sequester and transporter), YhdW (membrane component of amino acid ABC transporter), HlyA (associated with biofilm formation), and a putative outer membrane exporter protein (herein referred as pOMP1). These proteins were seen to primarily interact with various human proteins belonging to the ABC transporter family and the V-type proton ATPase subunit B (ATP6V1B2). ATP6V1B2 which plays a crucial role in phagosomal maturation was found to interact with FlgC (flagellar basal body protein) and EivG (type III secretion apparatus protein) in all strains of *E. coli* O157:H7, but was found to be absent in the interactome data pertaining to MG1655 (the non-pathogenic *E. coli* strain used in this study). Considering the vital role of type III secretion system in the virulence of several gram-negative bacteria, it could be assumed that this HPI may have a significant effect of the manifestation of pathogenesis by the *E. coli* O157:H7 strains.

The host protein ABCA12 was predicted to interact with ChuT and YhdW in all the pathogenic *E. coli* strains. ABCA12 has earlier been reported to be associated with lipid transport mechanisms, especially for the transport of glucosylceramides (Wallrodt et al., 2013). Notably, early stages of entericinfection, caused by gram-negative bacteria like *Shigella*, have been shown to be dependent on accumulation of cholesterol and sphingolipids, like glucosylceramides (Lafont et al., 2002). Given that the pathogenic factors (and the virulent mechanism) of pathogenic *E. coli* bear high resemblance to that of *Shigella*, the set of HPIs involving human ABCA12 assumes significance. Similar to ABCA12, the human protein ABCB1 was also found to interact with ChuT as well as YhdW in all the pathogenic *E. coli* strains. YhdW is a component of ABC transporter and biosynthesis of LPS in *Escherichia* and is known to be mediated by an ATP-binding cassette (ABC)-transporter-dependent mechanism (Greenfield and Whitfield, 2012). Further, an earlier study had reported the involvement of lipopolysaccharide (LPS) in adhesion, retention and transport of *E. coli* JM109 (Abu-Lail and Camesano, 2003). Moreover, a recent study has reported the role of human ABCB1 in minimizing the bacterial adhesion to the gastrointestinal cells (Crowe and Bebawy, 2012). Thus, the predicted HPI between human ABCB1 and bacterial proteins may be crucial for deciding the fate of bacterial attachment to the host cell surface. Literature evidence supporting this small subset of HPIs (which are unique to the interactome data of the studied pathogens) suggest that these HPIs may be regarded as specialized adaptations of the pathogenic *E. coli* strains. These HPIs probably conform a competitive edge to the pathogenic strains of *E. coli* (over the non-pathogenic strains) and may thus be implicated for their virulent nature.

#### Human–*E. coli* protein interactions specific to non-pathogenic strain

The interactome data pertaining to the HPIs of the non-pathogenic *E. coli* strain (MG1655) was seen to harbor a subset of 45 HPIs which were found to be absent for the pathogenic strains (Figure 1). Bacterial proteins like Fe(3+) dicitrate transport protein (FecA), Fe(3+) dicitrate-binding periplasmic protein (FecB), primary amine oxidase (TynA), and D-allose-binding periplasmic protein (AlsB) were predicted to be involved in interactions with several human proteins including Ribulose-phosphate 3-epimerase (RPE), Antigen peptide transporter *2*(TAP2), and a host of aldehyde dehydrogenases and ABC transporters. In complementation to the *fhu* operon, MG1655, like most other commensal and laboratory strains of *E. coli*, was observed to possess the *fec* operon, an additional mechanisms for iron uptake. FecA and FecB (members of the *fecABCDE* ferric citrate transport system) in MG1655 was seen to interact with several host (human) cell transporters including, ABCA10, ABCG8, ABCB4, ABCC6, and ABCC8. In yet another PPI that is specific to the non-pathogenic strain, AlsB, the periplasmic substrate-binding component of the allose ABC transporter, was found to interact with human RPE. It was interesting to note that the *alsBAC* system, though primarily meant for allose transport, could also function as a low affinity ribose transporter (Kim et al., 1997). The above observations lead us to hypothesize that the HPI involving AlsB and RPE in *E. coli* MG1655 is probably involved in uptake of additional nutrients to sustain its growth in a nutrient-depleted environment.

TynA, the copper-containing amine oxidase, was observed to interact with host alcohol dehydrogenases. Although, previous studies involving *E. coli* amine oxidase could not establish the role of TynA in pathogenesis, TynA activity (coupled with H_2_O_2_ production) has been demonstrated for growth under stringent environmental conditions (Murooka et al., 1978; Elovaara et al., 2015). Furthermore, the potential of TynA to utilize certain (unknown) substrate on human leukocytes have also been solicited (Elovaara et al., 2015). The observed PPIs between host alcohol dehydrogenases and bacterial TynA assume importance and require a deeper probe in this regard.

Overall, the non-pathogenic strain of *E. coli* was seen to be involved in a higher number of HPIs as compared to the pathogenic strains. Although the exact reason behind the existence of this added repertoire of HPIs in non-pathogenic *E. coli* remains unclear, it is likely that this subset of HPIs facilitates the non-pathogenic strain of *E. coli* (MG1655) in optimally utilizing the resources within the nutrient-depleted environment of the host gut.

#### Lineage and strain specific human–*E. coli* protein interactions

A subset of inter-species PPIs was predicted to be specifically present in the interactome data pertaining to certain lineages of *E. coli*. In one such case, YbeQ was observed to interact with several human proteins including derlin-1 (DERL1), protein OS-9 (OS9), E3 ubiquitin-protein ligase synoviolin (SYVN1), and ubiquitin-conjugating enzyme E2 J1 (UBE2J1). Interestingly, these host proteins are known to be functionally linked to the endoplasmic reticulum-associated protein degradation (ERAD) pathway. ERAD ensures that only the correctly folded proteins are secreted by the host (human) cells. Some of these secreted proteins (whose correct folding conformations are ensured by ERAD) may be detrimental to the invading pathogen. Inspection into the *E. coli* YbeQ protein sequence revealed the presence of repetitive SEL-1 domain. Evidence from literature suggests that (eukaryotic) proteins containing SEL-1 domain(s) often influences the mechanism of ERAD (Neuber et al., 2005). Consequently, the interaction of bacterial YbeQ with the host protein involved in ERAD seems to be significant and requires further investigation. This subset of interactions was observed in the host–pathogen interactome data corresponding to only two (EDL933 and Sakai) out of the four *E. coli* O157:H7 strains as well as in *E. coli* K-12 strain. Interestingly, EDL933 and Sakai are members of lineage I of *E. coli* O157:H7 which are known to be better equipped in colonizing the human gut as compared to the other lineages of *E. coli* O157:H7 (Lowe et al., 2009). Similar HPIs, specific to members of lineage I of *E. coli* O157:H7, involving a poorly characterized bacterial ferric transporter protein (herein referred to as ucp1) and host proteins like ABCA12, ABCB1, ABCB4, and ABCB5 were also observed. Such HPIs suggest possible roles of YbeQ and ucp1 in manipulating the host immunity and/or metabolism, thereby aiding in preferential colonization of the human gut. Furthermore, a HPI involving bacterial FdoG (α subunit of formate dehydrogenase O) and human EEF1A2 (Elongation factor 1-alpha 2) was observed to be unique to *E. coli* TW14359 strain. Interestingly, FdoG mutant *Escherichia* and *Salmonella* have previously been shown to be susceptible to human bactericidal/permeability-increasing proteins (BPIs) during stationary phases of growth (Barker et al., 2000). This underlines the key role of bacterial FdoG in evading host defenses, thereby leading to sustained enteric infection. However, the specific mechanism as well as consequence of the interaction between EEF1A2 and FdoG is not clear.

Inspite of its non-pathogenic nature, *E. coli* MG1655 was seen to share at least six PPIs with *E. coli* EDL933, a pathogenic strain of lineage I, which was found to be absent in one or more of the other studied pathogenic strains. Of them, the interaction between bacterial YcjM and human PYGB was found to be absent in all except *E. coli* MG1655 and EDL933 strains. Interestingly, PYGB has been seen to be up-regulated in studies pertaining to pulmonary infections caused by *Burkholderia cepacia* (Mariappan et al., 2013). The study however have not introspected the probable reason behind such observation. Given the key role of PYGB in glycogen metabolism, the observed HPI may have significant bearing on the host metabolism. Thus, our finding opens up an opportunity to further investigate into the mechanism of HPIs involving human PYGB.

Interaction among *E. coli* UidC and human GUSB is yet another example of a HPI that was observed to be selectively present in host–pathogen interactome data pertaining to *E. coli* MG1655, EDL933, and TW14359. The UidC in *E. coli* is a component of the *uidABC* system, which is involved in transport and utilization of glucuronides. It is pertinent to note that steroid hormones, pharmaceuticals, toxins, xenobiotics, etc. are often excreted by the host (human) liver through bile as conjugated glucuronides. These glucuronides may be deconjugated by bacterial glucuronidases (like UidA), to release the apolar aglycone which in turn, may be utilized by the bacteria as carbon source (Liang et al., 2005). Notably though, the *uidABC* system is known to be non-functional in MG1655 due to a mutation in the *uidB* gene (Liang et al., 2005). These subtle differences which were observed in the HPI profiles among the different strains of *E. coli* may probably be attributed to evolutionary events like speciation, horizontal gene transfer, gene loss, as well as periodic insertion-deletion-substitution of nucleotides, thereby leading to lineage and/or strain specific variations.

### Understanding the role of human and *E. coli* proteins during infection

**Figure 2:**
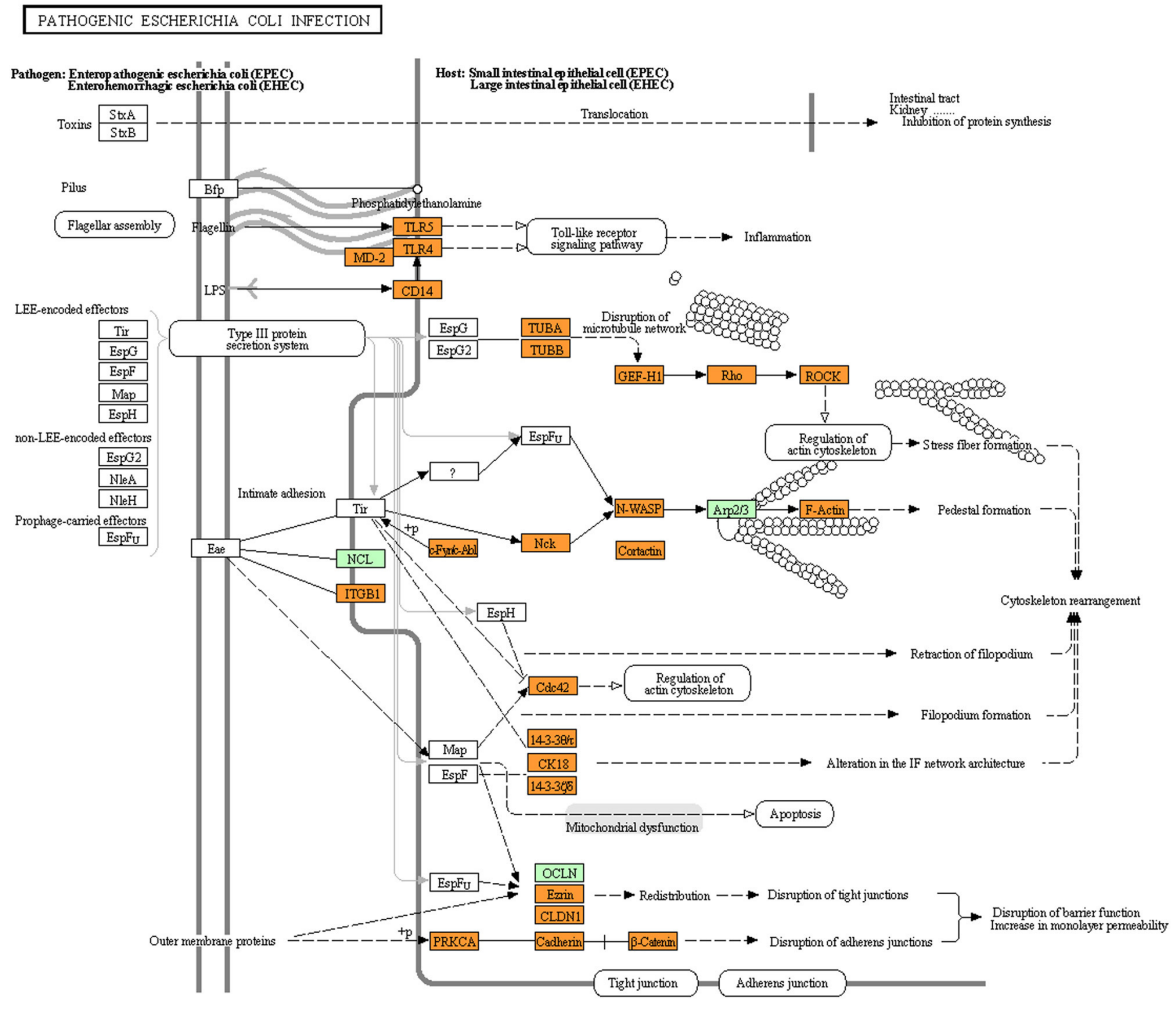
**Visualization of the KEGG pathway for “pathogenic ***Escherichia coli*** infection” overlayed with the human proteins (highlighted in orange) that were closely associated with the human–***Escherichia coli*** PPI networks**. Proteins highlighted in green represent human proteins (from the KEGG “pathogenic *Escherichia coli* infection” pathway) which were not closely associated with the human–*Ecsherichia coli* PPI network.

The analysis of the intra-species PPIs involving different strains of *E. coli* revealed that the majority of the PPIs are common to both the pathogenic as well as the non-pathogenic strains. Only a small subset of PPIs was predicted to differentiate the pathogenic strains from the non-pathogenic ones. It would therefore be interesting to understand whether this small subset of interaction plays a role in the manifestation of *E. coli* infection process. The KEGG pathway for infections caused by pathogenic *E. coli* (ko05130), enlists the human proteins which are probably involved in the virulence process. We investigated the human proteins, which are reported (in the KEGG database) to be involved in the *E. coli* infection machinery (referred to as set-B in the Materials and Methods Section) to check whether they are connected to the host (human) protein associated to PPIs with *E. coli* (referred to as set-A in the Materials and Methods Section). We observed that majority of the proteins of human origin, which were reported (in KEGG) to be affected by the *E. coli* pathogenesis, were closely related to the human proteins participating in PPIs (Figure 2). Surprisingly though, for all the five studied *E. coli* strains, the list of human proteins which were predicted to take part in PPIs, and were also closely associated with the proteins involved in *E. coli* infection machinery (as reported by KEGG) was identical. This probably indicate that the small subset of PPIs which were specific to either of the pathogenic or the non-pathogenic strains do not play a direct role in the *E. coli* infection machinery. However, given that most studies related to *E. coli* are based on the laboratory cultured strains (like *E. coli* MG1655), it is probable that our observation is an effect of the lack of proper understanding of the *E. coli* infection machinery. While human proteins (reported in ko05130) like CDC42, CD14, PRKCA, TLR4, etc. were directly connected (first degree neighbors) to one or more of the host proteins participating in PPIs, others proteins reported in the KEGG pathway for *E. coli* virulence (such as, NCK2, LY96, ABL1, and CDH1) were observed to be close neighbors (second degree connections) of the interacting host proteins (data not shown). Some of the human proteins (viz. NOS3, MAPK14, TNF, NFKB1) which were predicted to be involved in PPIs, were observed to be directly linked (first degree connection) to multiple host proteins from the KEGG *E. coli* virulence pathway (ko05130) including RHOA, CD14, PRKCA, TLR4, and CDC42. Notably, the mechanism by which the *E. coli* proteins influence the functionality of the human proteins (as reported in ko05130) is not clearly known. For example, although it is known that the *E. coli* proteins are involved in altering the host processes like cytoskeletal rearrangement, microtubule destabilization and programmed cell death (apoptosis), the mechanistic aspect of how the components of the type III secretion system transmute these biological processes of the host is poorly characterized. The above observations motivated us to inspect into the possible molecular mechanisms that may be involved in the cellular signaling processes that are triggered in response to the pathogenic *E. coli* proteins.

It is evident from KEGG's Pathogenic *E. coli* Infection pathway (ko05130), that there is an inter-play between the flagellar assembly/biogenesis process of EHEC and the human Toll-like receptor signaling pathway, which finally leads to host inflammatory responses. Signaling through the Toll-like receptor signaling pathway is triggered by TLR4 and TLR5 (Figure 2). However, the mechanism of action is not clearly understood. Our analysis revealed that the human TLR4 is functionally linked to MAPK14, which in turn, interact with the pathogenic DegS (Supplementary Table 1). The bacterial DegS is a member of the DegU-DegS two component system which is known to be associated with flagellary movement. A recent study on *Bacillus subtilis*, has reported that the inhibition of flagellar rotation functions acts as a mechanical trigger for the activation of the DegU-DegS two-component signal transduction system (Cairns et al., 2013). Once the pathogenic *E. coli* attaches to any surface (such as, the human intestinal epithelial cell surface), the flagellary motor movement is halted, thus triggering DegU-DegS system and thereby allowing DegS to interact with the human proteins. This indicates that the mechanism of activation of the host Toll-like receptor signaling pathway is possibly mediated through the interactions between the *E. coli* DegS and the human MAPK14 and TLR4 proteins. It is also interesting to note that DegU-DegS is also responsible for the biosynthesis of the exopolymer poly-γ-dl-glutamic acid, which is associated with immune evasion and virulence in *Staphylococcus epidermidis* (Kocianova et al., 2005). Furthermore, TLR4 and TLR5 are also observed to interact with CDC42, a protein involved in regulation of actin cytoskeleton assembly. Given this role of CDC42 in microtubule rearrangement, a probable role of DegS in mediating phagocytosis may also be speculated.

The most common symptom of diseases caused by the enteropathogenic *E. coli* is watery diarrhea. Disruption of tight junctions and monolayer permeability leading to loss of fluids from epithelial cells has been reported to be the cause of diarrhea. E-cadherin and its interaction with beta-catenin (CTNNB1) are known to regulate the monolayer permeability of the host cells. It has been reported that once the pathogenic *E. coli* adhere to the host epithelial cells, its outer membrane proteins (OMPs) dissociate the cadherin/beta-catenin complex, thereby leading to watery diarrhea (Malladi et al., 2004). Adhesion of *E. coli* to the epithelial cell membrane is however a pre-requisite for the cause of any such clinical symptoms. Mannose and its derivatives like mannose-6-phosphate are known to inhibit the process of cellular adhesion by the piliated strains of *E. coli* to the mammalian epithelial cells (Ruggieri et al., 1985). In this regard, it is pertinent to note the involvement of pathogenic phosphomannose isomerase (PMI/ManA) in HPI with the human proteins. ManA, which is secreted by *E. coli*, catalyzes the conversion of mannose-6-phosphate and fructose-6-phosphate, thereby, allowing the *E. coli* to adhere to the host epithelial cells (Ruggieri et al., 1985). In addition, in studies relating to human breast cancer cell lines, phosphoglucose isomerase (PGI), which is a functional analog of ManA/PMI, has been seen to down regulate E-cadherin/beta-catenin complex formation through inhibition of E-cadherin expression and enhancement of proteosomal degradation of beta-catenin (Wu et al., 2008; Funasaka et al., 2009; Jia et al., 2013). These observations indicate that the bacterial ManA/PMI may play a very crucial role in pathogenesis. The above evidence seems to suggest that in addition to aiding the bacteria to adhere to the host cell surface, ManA may also aid the pathogen in disrupting the tight junctions within the host epithelial cell lining, thereby resulting in watery diarrhea.

### Potential of *E. coli* to colonize within the human gut

HPIs play a critical role in the survival and sustenance of external agents like viruses and bacteria inside a host system. The HPIs help the invading pathogen to adhere to the host cell surface and subsequently to counter the host's immune responses. In addition, in cases of intracellular pathogens like *Mycobacterium tuberculosis*, the HPIs also play part in facilitating the phagocytic uptake (by host immune cells) and intra-cellular survival of the pathogen. Notably, it was observed that the majority of inter-species PPIs were ubiquitous to all the studied strains of *E. coli*, irrespective of their pathogenic nature. Further, the human proteins involved in PPIs and those which are closely associated with the *E. coli* infection machinery (as reported in KEGG database) were mostly similar for all the studied strains of *E. coli*. Given this, the gene expression profiles of the human and *E. coli* proteins which were involved in HPIs were studied to estimate the proportion of proteins which may be involved in these interactions. It was assumed that a ratio of cumulative score of bound protein to total protein concentrations (involved in different PPIs) would indicate the extent of colonization. Based on this assumption, a metric to determine the host colonization potential of the *E. coli* strains was designed. This metric was termed as the HCI. The procedure of deriving the HCI metric has been described in the Materials and Methods Section.

It was observed that host-associated stains of *E. coli* had higher HCI-values (>0.99) as compared to the non-pathogenic laboratory strain (0.823), which is indicative of the superior potency of host-associated (pathogenic) strains to colonize the human gut Furthermore, it was observed that strains of both lineages I and lineages I/II had comparable HCI-values (0.996 and 0.997, respectively). It may be noted that strains of lineages I (EDL933 and Sakai) and lineages I/II (EC4115 and TW14359) had fewer number of PPIs as compared to laboratory (or environmental) strains. This indicated that HCI is not dependent on the total number of PPIs but is a function of the amount of proteins that are produced in the cell and are involved in the PPI events. Thus, host–pathogen pair which share a fewer number of interactions, can potentially have a relatively higher HCI as compared to a host–pathogen pair sharing a larger array of PPIs, and subsequently a better colonization potential. Pertinently, unlike gram-negative bacteria such as *E. coli*, the gram-positive bacteria possess a thicker cell wall and harbors a significantly lower number of outer membrane vesicles. Hence, in the case of gram-positive bacteria, a fewer number of surface (and secreted) proteins are expected to take part in interaction with the host. HCI, being independent of the total number of PPIs, is aptly suited to quantitatively portray the potency of a pathogen to successfully survive within the host.

### Comparison between host–*E. coli* interaction patterns in humans and cattle

*E. coli* O157:H7 is primarily known to colonize the ruminant gastrointestinal tract (Zhang et al., 2010). Interestingly though, while human infected with *E. coli* O157:H7 suffers from several enteric diseases, cattle infected with *E. coli* O157:H7 appear to be mostly asymptomatic (Karmali, 1989; Karch et al., 2005; Zhang et al., 2007). As a result, some of the earlier studies were focused on identifying the spatiotemporal nature of immune response in cattle when infected with *E. coli* O157:H7 (Tildesley et al., 2012). In an attempt to get better insights into the basis of the variations in immune responses of humans and cattle on exposure to *E. coli* O157:H7 infection, analysis of the HPI profiles of both humans and cattle was carried out. For this purpose, the profiles pertaining to HPIs between cattle and the five studied strains of *E. coli* were obtained using a strategy similar to that adopted while predicting HPIs between humans and *E. coli*. Homologs between the human and cattle proteins were determined using a BLASTp analysis (e-value ≤ 1 × e^−10^, identity ≥60% and minimum overlap of 80%). Supplementary Figure 1 presents a comparative analysis of HPI profiles of humans and cattle pertaining to the five studied strains of *E. coli*. Overall, humans seemed to be involved in a higher number of HPIs with *E. coli* than cattle. Further, in spite of certain commonalities among the HPI profiles of the two hosts, the majority of the interactions of human and cattle (with each of the studied strains of *E. coli*) were largely different. Given that in most cases cattle infected with *E. coli* O157:H7 are phenotypically asymptomatic, the above observation is important. A deeper probe into the functional role of the *E. coli* proteins involved in these differential HPIs among humans and cattle could help in understanding the basis of infections caused in humans.

In order to ascertain the exact role of the *E. coli* proteins which were observed to participate in HPIs with the host, a Gene Ontology (GO) Enrichment Analysis of these proteins (and their first degree neighbors) was performed. Overall, biological processes associated with transport of nutrients like ion transport (GO:0006811), amino acid transport (GO:0006865), carboxylic acid transport (GO:0046942), organic acid transport (GO:0015849), siderophore transport (GO:0015891), etc. were found to be enriched in all the sets of *E. coli* proteins involved in HPIs (irrespective of the associated host), suggesting the need for *E. coli* to acquire nutrients from its host for its survival and proliferation. GO terms were further inspected to identify biological process which was unique to the *E. coli* protein involved in HPI with humans and cattle (Table 1). Notably, the *E. coli* proteins associated with HPI in human were uniquely enriched in carbohydrate metabolic process terms. These included monosaccharide metabolic process (GO:0005996), cellular carbohydrate metabolic process (GO:0044262), glycolysis (GO:0006096), hexose metabolic process (GO:0019318), monosaccharide catabolic process (GO:0046365), and cellular carbohydrate catabolic process (GO:0044275). In contrast, *E. coli* proteins associated with HPI in cattle were uniquely enriched in nucleotide and nitrogen metabolic process terms, such as, nucleotide biosynthetic process (GO:0009165), nucleobase metabolic process (GO:0009112), purine salvage (GO:0043101), nitrogen compound biosynthetic process (GO:0044271). It is pertinent to note that earlier literatures have pointed at the vital role of gut micro-flora in production of amino acids (and subsequent protein assimilation) inside the rumen gut (Genzebu and Tesfay, 2015).

**Table 1:** **Uniquely enriched Gene Ontology (GO) terms corresponding to the proteins from the five studied strains of ***Escherichia coli*** which participated in PPI with humans and cattle**.

**GO terms associated with *E. coli* proteins specifically interacting with human**	**GO terms associated with *E. coli* proteins specifically interacting with cattle**
GO:0005996—monosaccharide metabolic process	GO:0055086—nucleobase, nucleoside, and nucleotide metabolic process
GO:0001539—ciliary or flagellar motility	GO:0034654—nucleobase, nucleoside, nucleotide, and nucleic acid biosynthetic process
GO:0048870—cell motility	GO:0009165—nucleotide biosynthetic process
GO:0044262—cellular carbohydrate metabolic process	GO:0009112—nucleobase metabolic process
GO:0006096—glycolysis	GO:0006163—purine nucleotide metabolic process
GO:0019318—hexose metabolic process	GO:0044271—nitrogen compound biosynthetic process
GO:0032774—RNA biosynthetic process	GO:0043101—purine salvage
GO:0046365—monosaccharide catabolic process	GO:0043174—nucleoside salvage
GO:0044275—cellular carbohydrate catabolic process	GO:0042946—glucoside transport
	GO:0015772—oligosaccharide transport

More interestingly, the human interactome components of *E. coli*, were also seen to be enriched in biological processes pertaining to ciliary or flagellar motility (GO:0001539), and cell motility (GO:0048870). Proteins associated with these processes were found to be absent in the HPI profile pertaining to cattle. Given that ciliary or flagellar motility functions may grossly be associated to virulence, this observation is particularly significant, especially in perspective of the fact that cattle harboring pathogenic strains of *E. coli* (like the O157:H7 strain) in their gut are mostly asymptomatic in nature. The human protein which were observed to interact with the *E. coli* proteins associated to ciliary and flagellar movement were seen to be involved in lipid transport (GO:0006869), sterol transport (GO:0015918) and transmembrane transport (GO:0055085) functions (data not shown). This is in concordance with the recent reports which highlighted the role of lipids residing on the host cell surface in flagella mediated bacterial adhesion to host epithelium (Rossez et al., 2014, 2015).

## Discussion

The current study encompasses the intra-species PPI data from human as well as different strains of *E. coli* to infer potential HPIs among the host and the pathogen proteins. Results indicate that the invading pathogens have devised ways to subvert the host's immune defenses, primarily by countering the redox stresses generated by the host. All the studied strains of *E. coli* were observed to possess an arsenal of genes including CysP, GadA, KatG, and KatE to neutralize the oxidative stress that are generated by the host's immune system in response to the pathogenic determinants in *E. coli*.

Different strains of *E. coli* were also observed to harness specialized repertoire of genes to acquire “scarce” nutrients from its environment. For example, FhuA and FhuD (members of the *fhu* operon) which were observed to interact with host ABC transporters are essential for iron acquisition for the pathogen under *in vivo* conditions. Certain non-pathogenic strains of *E. coli* (like *E. coli* K-12 MG1655) encoded for additional mechanisms of iron uptake through the ferric citrate transport system (*fec* operon). Furthermore, it was also interesting to note the selective presence of *uidABC* system in a few strains of *E. coli*. The *uidABC* system is involved in the deconjugation of glucuronides and subsequent uptake of the apolar aglycone by the bacteria to be used as carbon source for its growth. The *uidABC* system of MG1655 is particularly fascinating in this regard. Although the genome of MG1655 encodes for all the genes constituting the *uidABC* system, the operon is non-functional due to a single mutation at position 100 of the *uidB* gene (Liang et al., 2005), implying that the *E. coli* K-12 MG1655 is unable to utilize the rich bounty of glucuronides that are available inside the host gut. This leads us to speculate that one or more of the glucuronides (in its aglycone form), which often include steroid hormones, pharmaceuticals, toxins, xenobiotics, etc., may be toxic for the bacteria and most strains of *E. coli*, including the K-12 strains lacking the capacity to detoxify the same.

Genomes of the pathogenic strains of *E. coli* were seen to encode for additional proteins which were involved in the infection process. For example, pathogenic strains of *E. coli* encoded for HylA and EivG which aided the pathogen in biofilm formation and secretion respectively. In addition, the pathogenic strains also possessed genes like YhdW which helped the pathogen in transporting lipopolysaccharide (LPS) to the cell surface. Notably, LPS is known to help the bacteria in adhering to the host epithelium (Abu-Lail and Camesano, 2003). Consequently, given the role of human ABCB1 in minimizing bacterial adhesion to the gastrointestinal epithelium (Crowe and Bebawy, 2012), the predicted PPIs involving YhdW and human ABCB1 assumes importance.

Interestingly, there were no differences among the set of human proteins which interacted with different strains of *E. coli* and were in close proximity to those proteins which are known to be affected by *E. coli* pathogenesis (from the KEGG database). It may be speculated that there are additional factors like altered gene expression, alternate gene splicing, and epigenetic modifications which play a decisive role during the pathogenesis process.

Further, a comparative analysis of the HPIs pertaining to different strains of *E. coli* showed lineage and clade specific differences. Analysis of the enriched GO terms corresponding to the *E. coli* proteins (which were involved in HPIs), revealed that members of *E. coli* O157:H7 lineage I were uniquely enriched in lipopolysaccharide biosynthetic process (GO:0009103), response to hydrogen peroxide (GO:0042542), response to reactive oxygen species (GO:0000302), etc. This is particularly significant in the light of the fact that previous literature suggests that the *E. coli* O157:H7 strains belonging to lineage I are better adapted to survive within the human gut as compared to the other strains of *E. coli* O157:H7 (Lowe et al., 2009). Furthermore, genes/proteins involved in the GABA dependent acid-resistance were observed to be specifically enriched in *E. coli* Sakai strain. The above findings indicate that different strains of *E. coli* O157:H7 have evolved (or acquired) certain strain/clade/lineage specific techniques to bypass the host defenses.

In spite of the minor variations in the HPI profiles, the different biological pathways that are associated with the human proteins interacting with the different strains of pathogenic and non-pathogenic *E. coli* were observed to be largely similar. Yet, we know that the virulent potential of these pathogenic strains are not the same. These lead us to speculate that there might be strain specific variations in the level of expression of some of the pathogenic genes/proteins. In this regard, it was interesting to note the levels of gene expression of the stress responsive genes among *E. coli* O157:H7 strains belonging to different lineages (Wang et al., 2009). For example, on exposure to peroxynitrate stress, while the *cysP* gene, was observed to be significantly over-expressed among members of lineage I/II (TW14359) as compared to those of lineage I (Sakai). However, no significant variations in gene expression profile were observed for the other stress responsive genes (which participated in PPI with the host proteins; Wang et al., 2009). This indicates that in spite of possessing a common arsenal of stress responsive genes, *E. coli* residing in different ecological niches is able to alter the expression of these genes to efficiently cope with the environmental stresses.

Given the importance of the PPIs in the colonization/survival of the bacteria inside the host, it was assumed that the amount of host and bacterial proteins which was involved in these interactions would be indicative of the colonization potential of the bacteria. Thus HCI, the novel metric for estimating host colonization potential has been proposed. Host-associated (pathogenic) strains of *E. coli* were seen to demonstrate higher HCI-values as compared to the laboratory (non-pathogenic) strain. Higher HCI-values of the O157:H7 strains are indicative of their superior potential to colonize the human gut. In comparison, strains belonging to different lineages of *E. coli* O157:H7 had similar HCI-values, indicating that in spite of the differences in their virulent potential, their ability to colonize the human gut is the same.

The identified HPI are expected to be helpful in deciphering the biological basis of the diseases caused by different bacterial pathogens including *E. coli*. Investigating time-series gene expression data for the host and pathogen protein involved in HPIs would be beneficial in understanding the chronology of events during infection. The study opens up the scope for identifying new therapeutic targets in enterohemorrhagic pathogens. It also provides the potential of developing host directed therapies which are highly solicited in this era of rapid emergence of drug-resistant traits among different strains of bacteria.

## Materials and methods

### Data acquisition and preprocessing

A pre-requisite for comparative analysis of HPIs pertaining to different strains of *E. coli*, was to identify orthologous groups of protein among the studied strains. In addition, computation of host-pathogen interacting pairs also necessitated the determination of homologous protein sequences in humans and *E. coli*. Consequently, the protein sequence information for the five studied strains of *E. coli* as well as that of human were downloaded from the NCBI database (ftp://ftp.ncbi.nlm.nih.gov/genomes/). The protein sequences from the five strains of *E. coli* were subsequently clustered by employing the BLASTClust program from the standalone BLAST package. A total of 6,884 unique clusters, each representing a distinct functional group, were obtained using threshold criteria of 95% identity and 80% overlap among the clustered sequences. The sequence identity and overlap criteria were obtained from previous literatures on protein clustering (Junjie et al., 2010; Finn et al., 2014). The homologous sequences between *E. coli* and humans were identified by BLASTp analysis. Human and *E. coli* proteins were considered to be homologous if they shared at-least 30% identity and 80% sequence overlap with an e-value lower than 1 × e^−10^.

**Figure 3:**
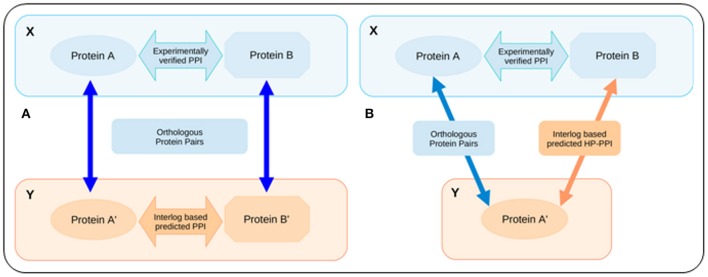
**Schematic representation of the interlogs approach**. **(A)** Interlog based mapping approach for predicting intra-species PPIs. If A–A′ and B–B′ are orthologous sets of protein in two organisms X and Y and the proteins A and B are known to interact in organism X, then the interlog based mapping approach concludes a possible PPI between the proteins A′ and B′ in organism Y. **(B)** Modified (inter-species) interlog method that was employed for predicting the HPIs. X represents the host and Y represents the pathogen (or vice versa). If protein A and protein B of organism X are known to interact among each other, and if there exists a homolog to protein A (protein A′) in organism Y, then the modified interlog method assumes that protein A′ will also interact with protein B through a PPI.

The intra-species interactome data for human and the five studied strains of *E. coli*, which were considered for creating the background library of template interactions, were downloaded from the STRING database, version 9.1 (http://string-db.org/; Franceschini et al., 2013). It is important to note that, in addition to the experimentally validated PPIs, the STRING database also collates PPI information using a wide range of techniques including text mining, homology determination, etc. PPIs determined by such non-validated techniques may often be spurious and are typically characterized by low combined confidence score. To minimize the false discovery rate of predicted HPIs, only the intra-species PPIs with a combined confidence score of 0.9 or higher were retained in the final set of template interactions. It is also pertinent to note that the STRING data classifies any given strain of bacteria as either “core” or “periphery.” It was observed that the PPI interactome data for the “periphery” strains were incomplete. Further, all the studied *E. coli* strains with the exception of *E. coli* MG1655 were found to be classified as “periphery” strains in the STRING database. Consequently, an interlog based mapping approach was put to use in order to obtain the complete interactome pertaining to all the five strains of *E. coli*. The interlog approach (Yu et al., 2004) is explained in the context of the current study in Figure 3A. The intra-species PPIs that were thus identified by this interlog mapping approach, were combined with the PPI data obtained from the STRING database (filtered with a combined confidence score of 0.9 or higher) to obtain the final set of template interactions.

### Identification of sub-cellular localization of proteins

Determining the sub-cellular localization of proteins is an important step in identifying HPIs. Sub-cellular localization of a protein governs its feasibility of participate in a HPI. For example, a pathogenic protein localized on the outer membrane or cell wall or one which gets secreted has a better chance of interacting with host proteins as compared to a pathogenic protein that is localized within the cytoplasm. Similarly, a membrane-associated or secreted host protein is more likely to interact with the proteins of an extra-cellular pathogen (like most species of *E. coli*). Since we were unable to mine the sub-cellular localization of most of the studied proteins from literature, we resorted to *in silico* prediction techniques. The sub-cellular localization of the bacterial proteins was ascertained using PSORTb (ver.3.0.2). PSORTb employs a signal sorting based search technique and was observed to outperform most of its competitors (Yu et al., 2010). Sub-cellular localization for the human proteins was obtained using WoLF PSORT, a widely used tool for eukaryotic protein sub-cellular localization prediction (Horton et al., 2007). The predicted sub-cellular localization of proteins from both the humans (host) and the five strains of *E. coli* (pathogen) were utilized in later steps for filtering the predicted (potential) HPIs.

### Prediction of host–*E. coli* protein–protein interactions

The PPIs were predicted using an approach similar to the interlogs method (Yu et al., 2004). It stated that if a PPI is known to exist among any two proteins in an organism and there are orthologs of those two proteins in any other organism, then those orthologous proteins should also participate in a PPI. HPIs (inter-species interlogs) were identified on the basis of the (intra-species) template interactions and the sequence homology data (that was obtained using the BLASTp analysis between human and *E. coli* proteins). In the inter-species interlogs method, a HPI was assumed to exist if (a) for a pathogen-specific (intra-species) template PPI, there is a homolog of at-least one of the interacting proteins in the host; or (b) for a host-specific (intra-species) template PPI, there is a homolog of at-least one of the host proteins in the pathogen. Figure 3B depicts a schematic diagram of the modified (inter-species) interlogs approach that was employed for determining the HPIs (inter-species interlogs). The set of potential HPIs (corresponding to the five studied strains of *E. coli*) thus identified were subsequently screened for the sub-cellular localization of the participating host and pathogen proteins. Only the subset of PPIs where both the host and the pathogen proteins were localized either on the cell membrane/cell wall or exterior to it, were considered to be legitimate host-pathogen interacting pairs. The above step resulted in the generation of five sets of host-pathogen interactome data, each corresponding to the HPIs predicted among humans and one of the five studied strains of *E. coli*.

### Host–*E. coli* interaction network analysis

The host–pathogen interactome data pertaining to the five studied strains of *E. coli* were analyzed for various network properties like degree and betweenness centralities of each node (constituent proteins in the network). For the purpose, the host–pathogen interactome data for each of the five strains of *E. coli* was analyzed using Cytoscape (version 2.8), a widely used platform for network analysis and visualization (Shannon et al., 2003). In addition, a comparative study of the five HPI networks was also performed. CompNet (Kuntal et al., 2016), a tool for comparative analysis of multiple biological networks, was employed for the purpose.

### Functional pathway analysis

The pathogenic *E. coli* infection pathway (ko05130) in the KEGG database enlists the human proteins which may be affected by the virulence mediated by pathogenic strains of *E. coli*. In order to determine the functional relationship among the set of human proteins that were predicted to interact with the pathogenic proteins (set-A) and the set of human proteins which were reported in the KEGG database to be affected by *E. coli* pathogenesis (set-B), the KEGG pathway analysis was performed. The human interactome data (as obtained from the STRING database) was filtered for a combined confidence score of 0.9 or higher. Based on the filtered human PPI data, the shortest paths connecting each of the components of set-A to those of set-B were determined. Shortest paths connecting the components of set-A and set-B with a path-length of two or less were retained. The shortest paths, thus determined, helped us in identifying the first and second degree connections between the set of human proteins that were predicted to interact with the pathogenic proteins and the set of human proteins which were reported in the KEGG database to be affected by *E. coli* pathogenesis.

### Computing host colonization index (HCI)

Host Colonization Index (HCI) was computed as the ratio of the cumulative function of bound:total protein concentrations (involved in different HPIs). First, the gene expression values for the host and *E. coli* proteins were obtained from NCBI GEO database^1^ (Gene Expression Omnibus; Maurer et al., 2005; Ukena et al., 2005; Lee et al., 2014). It was assumed that the gene expression values would be roughly proportional to the protein expression values and were considered as total concentrations (*Pt*) of the proteins. Next, for each of the proteins (*P*) which participated in HPI, the PPI module of the Steady State Gene Expression Simulator (SSGES; Srinivasan and Venkatesh, 2014) was employed to estimate the amount of host and pathogen proteins that may be bound to (interacting with) other proteins (*Pb*). Finally, HCI was computed as a ratio of the cumulative functions of bound:total protein concentrations for each of the host and pathogen proteins involved in HPIs.

$$\begin{array}{l} HCI=\;\sum \frac{Pb}{Pt} \end{array}$$

### GO enrichment analysis

The Gene Ontology (GO) enrichment analysis was performed using the Database for Annotation, Visualization and Integrated Discovery (DAVID) Functional Annotation Chart tool (Huang et al., 2009a,b). GO term enrichment analysis (level 4 BP terms) of the *E. coli* proteins involved in HPIs was performed with the following threshold cut-offs: count ≥2; and *p* < 0.01 (Bonferroni corrected).

## Author contributions

TB, KV, and SM conceptualized the work and the design of experiments. TB optimized critical parameters for conducting the experiments with inputs from KV and SM. TB performed the experiments, analyzed the results, and wrote the paper. KV and SM edited the paper.

## Funding

This work was partially supported by grants from the Department of Biotechnology (DBT), Government of India (BT/PR3260/BRB/10/967/2011).

### Conflict of interest statement

The authors declare that the research was conducted in the absence of any commercial or financial relationships that could be construed as a potential conflict of interest.

## Footnotes

^1^NCBI GEO database. Available online at: http://www.ncbi.nlm.nih.gov/geo/ (Accessed March 16, 2016).

## Supplementary material

Supplementary Table 1 **List of the predicted inter-species PPIs involving human proteins and those from the five studied strains of ***Escherichia coli*****.

Supplementary Figure 1 **Comparison analyses of the inter-species PPI patterns in humans and cattle involving proteins from different ***Escherichia coli*** strains**.
