# Supplementary figures and images for: Computational Analysis of Host–Pathogen Protein Interactions between Humans and Different Strains of Enterohemorrhagic Escherichia coli
Source: Front Cell Infect Microbiol. 2017 Apr 19;7:128. doi: 10.3389/fcimb.2017.00128 (PMC5395655; doi:10.3389/fcimb.2017.00128)

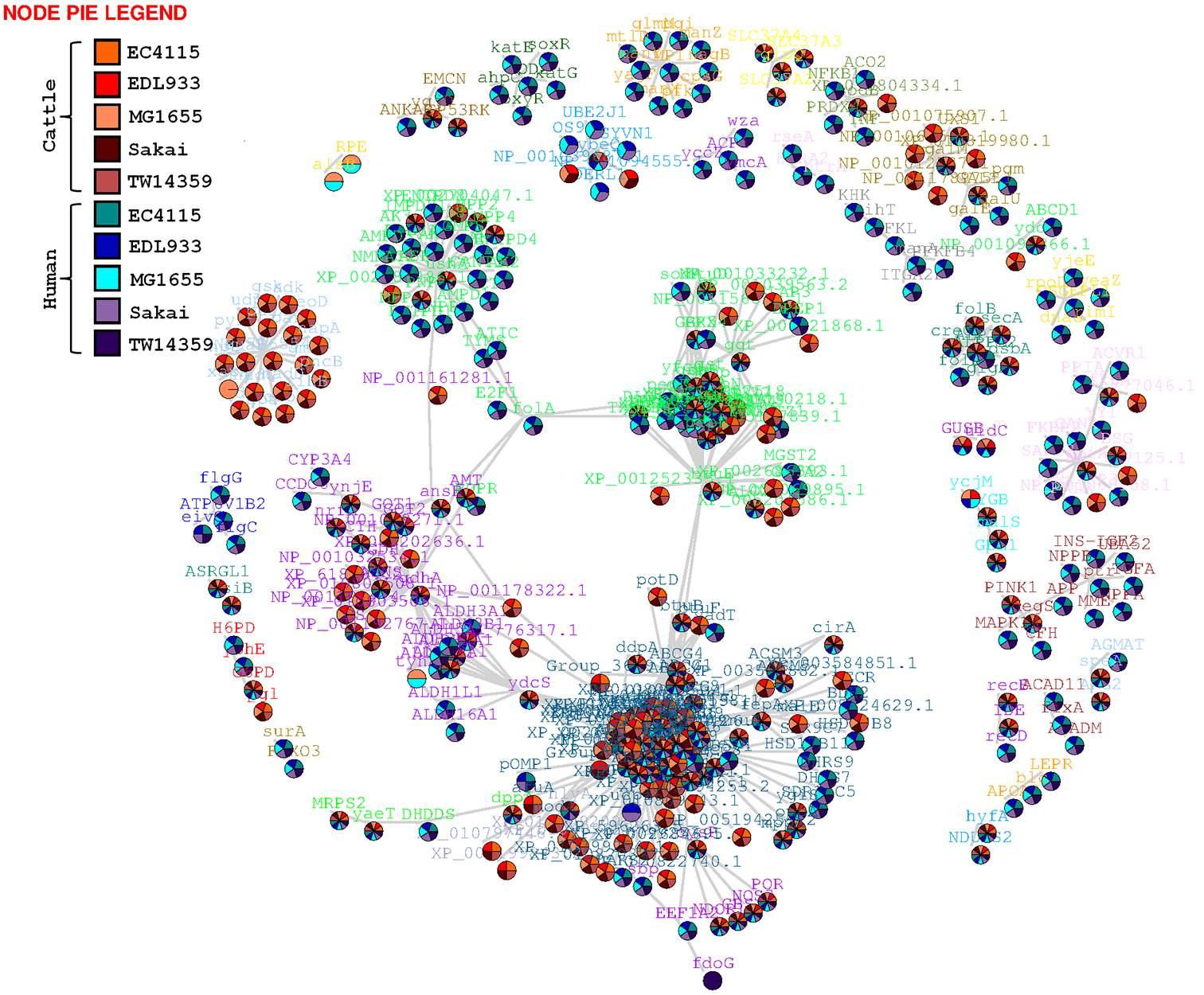

Supplement: Supplementary Figure 1 — Comparison analyses of the inter-species PPI patterns in humans and cattle involving proteins from different Escherichia coli strains. [file Image1.TIF]
